# Towards a fully automated algorithm driven platform for biosystems design

**DOI:** 10.1038/s41467-019-13189-z

**Published:** 2019-11-13

**Authors:** Mohammad HamediRad, Ran Chao, Scott Weisberg, Jiazhang Lian, Saurabh Sinha, Huimin Zhao

**Affiliations:** 1 Department of Chemical and Biomolecular Engineering, University of Illinois at Urbana-Champaign, Urbana, IL 61801 USA; 2 Carl R. Woese Institute for Genomic Biology, University of Illinois at Urbana-Champaign, Urbana, IL 61801 USA; 3 Department of Biochemistry, University of Illinois at Urbana-Champaign, Urbana, IL 61801 USA; 4 Department of Computer Science, University of Illinois at Urbana-Champaign, Urbana, IL 61801 USA; 5 Departments of Chemistry and Bioengineering, University of Illinois at Urbana-Champaign, Urbana, IL 61801 USA; 6 Present Address: LifeFoundry Inc., 60 Hazelwood Dr., Champaign, IL 61820 USA; 7 Present Address: Key Laboratory of Biomass Chemical Engineering of Ministry of Education, College of Chemical and Biological Engineering, Zhejiang University, 310027 Hangzhou, China

**Keywords:** Microbiology techniques, Metabolic engineering, Synthetic biology

## Abstract

Large-scale data acquisition and analysis are often required in the successful implementation of the design, build, test, and learn (DBTL) cycle in biosystems design. However, it has long been hindered by experimental cost, variability, biases, and missed insights from traditional analysis methods. Here, we report the application of an integrated robotic system coupled with machine learning algorithms to fully automate the DBTL process for biosystems design. As proof of concept, we have demonstrated its capacity by optimizing the lycopene biosynthetic pathway. This fully-automated robotic platform, BioAutomata, evaluates less than 1% of possible variants while outperforming random screening by 77%. A paired predictive model and Bayesian algorithm select experiments which are performed by Illinois Biological Foundry for Advanced Biomanufacturing (iBioFAB). BioAutomata excels with black-box optimization problems, where experiments are expensive and noisy and the success of the experiment is not dependent on extensive prior knowledge of biological mechanisms.

## Abstract (web summary)

Existing efforts have been focused on one of the elements in the automation of the design, build, test, and learn (DBTL) cycle for biosystems design. Here, the authors integrate a robotic system with machine learning algorithms to fully automate the DBTL cycle and apply it in optimizing the lycopene biosynthetic pathway.

## Introduction

Biological systems such as proteins, pathways and whole cells have been increasingly explored for a wide variety of biotechnology applications^1,2^. However, due to the complexity of biological systems and their myriad components and many unknown interactions among them, many rounds of design, build, test and learn (DBTL) must be performed^3–6^. There have been many efforts to expedite the DBTL cycle^3^ and automated biofoundries such as Illinois Biological Foundry for Advanced Biomanufacturing (iBioFAB)^7^ and Edinburgh Genome Foundry^8^ have been an undeniably important leap toward automating the design, build and test components of the cycle^3^. However, other than some specific and narrow applications^9,10^, there is no example of automation and integration of the learn component to close the DBTL cycle and enable the iteration of this cycle with minimal human intervention.

Furthermore, the automation is not limited to build and test elements of the cycle and given the large amount of data generated by modern biofoundries, automation of the learn component is also crucial. Assistance from computer algorithms and using statistical models and machine learning is of special importance given the complexity of most biological systems of practical importance and the high dimensionality of optimization tasks required to quantify and manipulate such systems. Biosystems ranging from single proteins to entire pathways can be engineered using statistical models^11,12^, machine learning algorithms^13–17^, reinforcement learning^18^ and a complete suite of biophysical models^19^. However, most of the progress on automation of the DBTL cycle has been focused on one of the elements of this cycle where integrating all these components can result in a synergistic effect of enabling large amount of high-dimensional data to be acquired and analyzed by the fully automated DBTL cycle.

To overcome these limitations, we integrate the iBioFAB, a fully automated and versatile robotic platform^7^ with a machine learning algorithm. This BioAutomata platform designs experiments, executes them and analyzes data to optimize a user-specified biological process in an iterative manner. BioAutomata trains a probabilistic model on initially generated (or available) data and decides the best points of the optimization space to evaluate, i.e., the points that are more likely to result in an improved biosystem. This results in a reduction of the total number of experiments needed to find the maximum of the optimization space. This optimization framework is ideal for cases where the goal is finding the optima of a black-box function and where data acquisition is expensive and noisy, which is intrinsically true in biosystems design. Bayesian optimization has also been shown to be a powerful tool in other areas such as protein engineering^15,16,20,21^.

As a proof of concept, we optimize the lycopene production pathway, i.e., fine-tune the expression of genes involved in its biosynthesis (the inputs to the function) to achieve the highest lycopene production (output of the function). Lycopene has been traditionally used as food additive and colorant but recently many reports have proposed its effects as antioxidant, anticarcinogen and for preventing cardiovascular disease^22^. Due to the high commercial value of lycopene, the lycopene biosynthetic pathway has been a target of multiple metabolic engineering pursuits^23–25^. While there are other strategies such as deleting or overexpressing endogeous genes in the organism to push the flux toward the product of the pathway, or simply optimizing the fermentation conditions, optimizing the expression of the biosynthetic genes is often the first choice. By combining the Bayesian optimization algorithm and iBioFAB automation system, we evaluate <1% of all the possible tunable expression values of component genes versus the production (expression–production landscape) to find a strain that produces high lycopene titer. Each point on this landscape denotes the production amount of the desired chemical given the particular expression level of each gene. After the initial design and setup of this BioAutomata, the role of the researchers changes from being the drivers of the experiments to supervisors of the system while the algorithm-driven optimization platform designs and performs the experiments to maximize the objective function defined by the researchers.

## Results

### Fully automated algorithm-driven platform BioAutomata

**Fig. 1:**
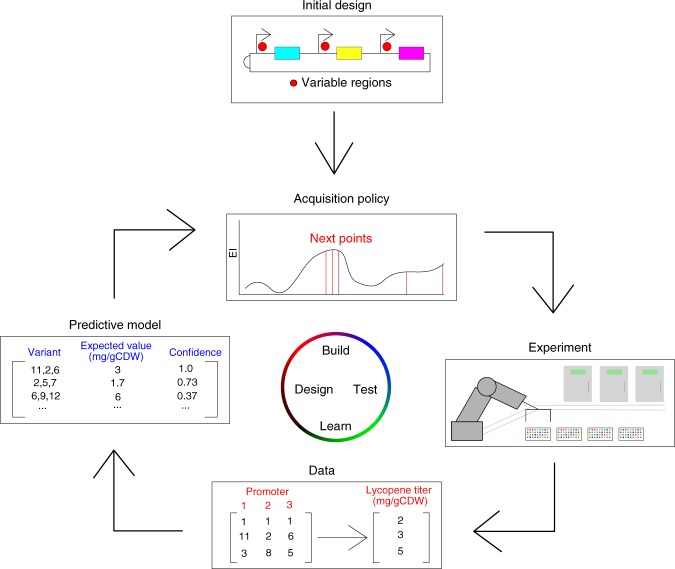
The overall workflow of BioAutomata. After setting the initial parameters, designing the sequence space of variable regions (such as promoter variants in a combinatorial pathway assembly), and defining the objective function, BioAutomata selects which experiments are expected to result in the highest improvement of yield, performs those experiments, generates data and learns from it, updating its predictive model given the newly presented evidence. It will then decide on the next experiments to perform to reach the goal set by the user while trying to minimize the number of experiments and the cost of the project

In biosystems design, it is typically expensive, time consuming and error-prone to perform wet-lab experiments. Therefore, optimizing a biological system is most efficient when the number of experiments performed is minimized. Our proposed approach to achieve this is shown in Fig. 1. Within this context, the first step in optimization is to determine the initial design, inputs and outputs of the system as well as the objective function. After the initial setup, a predictive model and acquisition policy should be chosen to estimate the landscape given the currently available data and choose the next points to be evaluated and experiments to be performed. After all the elements of the system (initial design, acquisition policy, experimental setup, data acquisition and predictive model) are chosen, the BioAutomata can commence the optimization. First, the acquisition policy chooses the points to be evaluated. Next, iBioFAB performs the experiments that evaluate the selected points for their fitness and returns the data to the predictive model. The model will then update its belief about the landscape based on the newly presented data. Last, the acquisition policy will choose the points to be evaluated next with the guidance of the updated predictive model.

### Determination of the predictive model and acquisition policy

Since the objective is to find the maximum of a black-box function where data acquisition is expensive and noisy, we sought to use Bayesian optimization^26^, which is ideal for solving such problems. Bayesian optimization^27–29^ is a powerful method that has been shown to outperform many algorithms^30^ in optimizing such challenging functions^31^. In short, it constructs a probabilistic model and uses this model to make decisions on where to evaluate next to maximize the expected progress made with each function evaluation and therefore reduce the number of evaluations, i.e., experiments required to find the maximum. The algorithm takes the expected outcome of each evaluation as well as the confidence on this expected outcome into account. To use this algorithm, two main functions must be chosen, a probabilistic model to make assumptions about the landscape given the available data and an acquisition policy to suggest which point to evaluate next to maximize the expected progress toward the optimum.

We used the Gaussian process (GP) as the predictive model to assign an expected value and confidence level to all the unevaluated points. The GP was chosen due to its flexibility and broad applications^15,30,32^. GP assigns a mean and variance to each point in the landscape and as more points are evaluated, the mean and variance are updated accordingly (Supplementary Fig. 1).

The acquisition function drives the experimental direction to make the most expected progress toward the optimum. Given the expected value and confidence on that value, we are faced with a trade-off between exploration and exploitation. If the only tested points are those with the largest expected values, we risk only finding local maxima. Hence, we want to explore more (focus on points where the model is uncertain about). However, if we only evaluate points where we have little confidence on the expected value, although we learn more about the landscape, in most cases these expensive experiments are wasted on increasing the confidence level on low-performing regions rather than focusing on finding the maximum. Hence, if we find a good point, we want to exploit that finding to search nearby for a better solution (with greater expectation).

Several algorithms are suggested for balancing the trade-off between exploration and exploitation and the maximum of acquisition function represents an automatic trade-off between these two factors. One of the commonly used acquisition functions is Expected Improvement (EI)^26,29^ where the algorithm estimates how much improvement over the current best is expected from each one of the points, and samples the point with the highest expected improvement. This function elegantly finds the balance in exploration and exploitation trade-off by using the already trained GP and finds the point that provides the highest expected improvement and was chosen as the acquisition function in this work.

As described before, by design, Bayesian optimization relies on sequential experiments. Each time one point is evaluated, the result is given to the algorithm to update the prior GP and find the next point to be evaluated using the acquisition function. However, it is more efficient to perform some experiments in parallel and in sequential batches so as to reduce the number of rounds of the experiment and consequently the time of the entire project. Fortunately, a variation of Bayesian optimization has been recently developed for multi-core parallel processing applications. This algorithm can handle multiple pending evaluations and can get the result of any of the pending evaluations at any given time and return the next point to be evaluated^26^. In short, the algorithm considers likely outcomes for each of the pending points and calculates the acquisition functions based on the all possible outcomes. This method was used to drive the direction of our experiments and one batch of points was chosen and evaluated in each round and the result was given to the algorithm to generate the next batch of points to be evaluated. It is noteworthy that in the experimental setting and when the evaluations are done using parallel experimentation, the pending points are updated at the same time in subsequent batches and not one by one.

If there was no error in the experiments, which is the case for evaluation of mathematical functions, the confidence level around the points that are already evaluated would be very high. However, since the result of all experiments contain some error and is far from perfect mathematical calculations, the confidence in the results was adjusted so the program expects an error in the evaluations and adjusts the mean and variance for all the points accordingly. The other aspects of this optimization algorithm including the covariance functions and hyperparameters of the GP are explained in details by Sneok and coworkers^26^.

### Evaluation of the Bayesian optimization algorithm

**Fig. 2:**
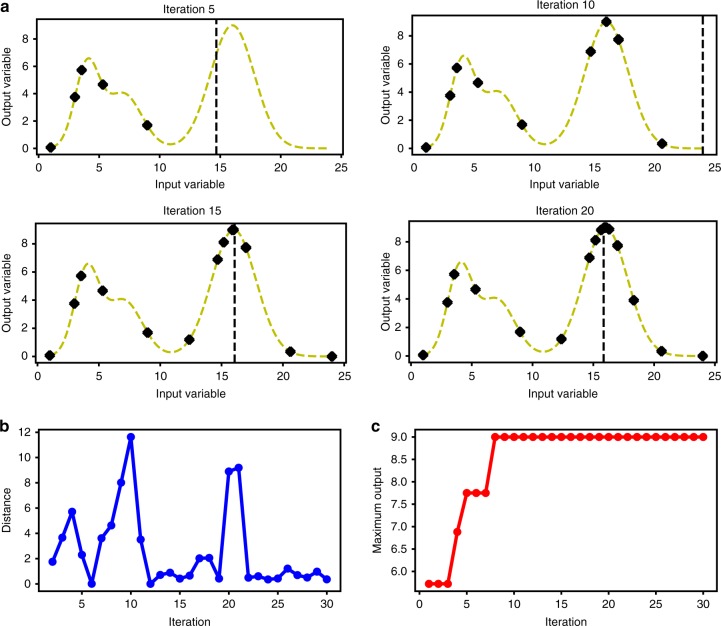
Testing Bayesian optimization by finding the maximum of a two-dimensional function. **a** The acquisition function decides the next input to test and the output is used to refine the predictive model. Iterations 5, 10, 15 and 20 of this process are shown. **b** With increasing rounds of iteration, the predictive model grows more confident of the location of the global maximum and the distance between tested inputs decreases with each iteration. **c** The algorithm evaluated 9 points before finding the location of the maximum. Subsequent iterations tuned this approximation toward the true optimum. The algorithm evaluated 12 points before finding the maximum. The order in which each point is evaluated is shown on the graph

To illustrate Bayesian optimization with GP, we defined a single variable function and tried to find the maximum value by sequential sampling (Fig. 2). The function was deliberately chosen to have multiple peaks and local optima (dashed curve in Fig. 2) to test whether the optimization algorithm can indeed find the global maximum. The algorithm was able to find the maximum and the exploration and exploitation trade-off is illustrated by the sampling order depicted in the figure. The more points evaluated by the algorithm, the closer the algorithm became to the maximum as shown in Fig. 2b.

We next sought to illustrate the optimization method with a similar 3-variable function with three inputs and one output to simulate a similar multi-dimensional optimization problem. It is noteworthy that Bayesian optimization has been used in numerous applications^33–37^ and the purpose of this simulation is testing the algorithm on a simple but similar setting. The search perimeter was set to be 1–24 for each of the inputs and the maximum of the function was set to be 9 (*y* *=* *f*(*x*_1_, *x*_2_, *x*_3_) | x_*i*_ *ɛ* {1, 2,…, 24}, *f*_max_ = 9). The Bayesian optimization algorithm was able to find the maximum value of this function by only evaluating 12 points out of all possible 24^3^ = 13,824 points. These 12 evaluations were the result of 12 iterations of learning and testing, with each evaluation being followed by a learn step that produced the next point to evaluate. We then sought to compare this optimization strategy with baseline approach where randomly sampled points are evaluated and all of these points are used to train an Exterior Derivative Estimation (EDE)-based regression model described in previous publications^14,38^. We found that although the EDE approach shows impressive predictive capability, especially given that all the data have been acquired at once and not through iterative sampling, even after sampling 192 random points, the maximum could not be found (Supplementary Table 1).

We then tested the Bayesian optimization method by running multiple simulations with different conditions. First, to see if the algorithm can find the maximum of other functions than the one tested in the previous section, we generated 100 random Gaussian mixture models and found the maximum for all of them using this algorithm. On average, it took the algorithm 9.82 and 7.93 evaluations to find the maximum and 95% of the maximum, respectively. To test the effect of error on the algorithm, we randomly picked one of these 100 Gaussian mixture models and attempted to find the maximum while adding 0%, 10% and 20% error rate, the upper bound of most analytical methods, to the output value of the function evaluation to better simulate the real experimental setup. We observed that the algorithm is still able to find the maximum of the function in most runs, but the number of evaluations in each run was significantly increased and in the case with 20% error, it could not find the maximum for 18% of the cases even after 400 evaluations. However, the algorithm could find 95% of the maximum in all cases (Table 1). This shows that, as expected, error makes the optimization more difficult, but Bayesian optimization algorithm can adjust for it and still find the maximum for most cases. It is noteworthy that finding the maximum gets increasingly difficult with higher error rate. Other than the fact that low-quality data, as expected, reduce the predictive power of the model, with higher error rate, the difference between points closest to the maximum becomes indistinguishable.

**Table 1:** Effect of error on the optimization

Points Before Max	Points Before 95%	Percentage Max Found	Percentage 95% of Max Found	Error
7.67	6.48	100%	100%	0%
57.23	22.66	100%	100%	10%
137.41	57.03	82%	100%	20%

Note: Higher error in evaluation of the objective function significantly impacts the performance of the maximization algorithm. Points Before Max represents the number of evaluations before maximum is reached, on average across 100 simulations, and Points Before 95% represents the average number of evaluations needed before reaching a point that is at least 95% of the maximum. Percentage Max Found and Percentage 95% of Max Found indicate how often, across the 100 simulations, the optimizer found the global maximum or a point that is at least 95% of the maximum. Finding the absolute maximum becomes increasingly difficult as the difference between points gets increasingly less distinguishable with higher error rate. Source data are provided as a Source Data file

Lastly, we set to optimize the number of points evaluated in each round, with a trade-off between the experimental cost and time: as the size of each batch increases, the cost of experiment increases as well, however, the number of total rounds of experiment, hence the time spent on the entire project decreases. The batch sizes are also constrained by experimental conditions especially given the standard 96-well format for high-throughput biological experiments. A few batch sizes were simulated on the test model described above while including 10% error. It was found that batch sizes larger than 46 did not significantly decrease the number of rounds in the given 4-D optimization scheme (Supplementary Table 2) and 46 was chosen as the batch size for pathway optimization experiments in this work.

### Automated optimization of the lycopene biosynthetic pathway

After finalizing the predictive model and acquisition policy, we chose optimization of the lycopene biosynthetic pathway as a model system. One of the reasons for the low productivity and yield of a biosynthetic pathway is flux imbalance^39–41^ where suboptimal reactions rates result in accumulation or depletion of the intermediates molecules in the reaction. This is especially important in pathways with multiple reactions where the intricate balance of each step of the pathway can be difficult to find. Fine-tuning the flux of each step in a pathway and its optimization has been shown to be a very effective strategy for increasing the total flux in a variety of different cases^42–45^. The abstraction of this problem can be represented by an expression–production landscape where the maximum flux is achieved by a certain expression level of each of the genes in the pathway. We should then design an experimental setup where we can tune the expression of the genes in the pathway (inputs of the function) and define the output that we want to maximize. We should then try different expression levels as inputs and get lycopene production as output and find the input that corresponds to the highest output.

To perform the expression tuning for pathway optimization and generating the inputs, a set of regulatory elements must be developed to control the expression level of the enzymes in the pathway of interest. Relying on previously published work, we mutated a region in T7 promoter that has been attributed to its strength^46,47^ to construct 12 promoters with distinct expression levels. We then designed and tested two Ribosome Binding Sites (RBS) using the RBS library calculator^40,45^ with vastly different strengths. The resulting T7p-RBS combination resulted in 24 distinct expression levels (Supplementary Fig. 2) with ~1000-fold dynamic range. To investigate  whether the expression level trend measured using eGFP translates to the trend with the *crtE*, *crtB*, and *crtI* genes downstream of the RBS, these genes were fused to eGFP and for each of the genes, four promoter/RBS combinations from four distinct combinations of weak/strong promoter/RBS, each randomly picked from one quartile or expression level strength, were compared and the same general expression trend was observed (Supplementary Fig. 3).

**Fig. 3:**
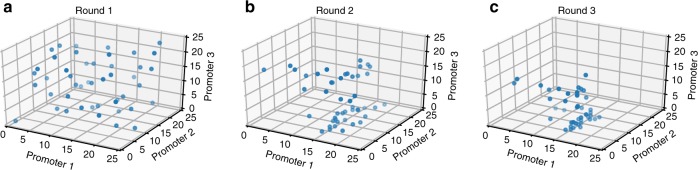
Change in sampling behavior of Baysesian optimization of the lycopene production pathway. In the first round (**a**), all points were chosen to uniformally explore the landscape since it is completely unexplored and unknown (*n* = 46). In the second round (**b**), some information is acquired and the points picked by the algorithm are clearly skewed from the unifrom distribution (*n* = 45). However, since there is some uncertainty, it is still exploring the landscape. Finally, in the third round (**c**), a clear pattern is observed where the algorithm has determined the points in a particular area are more likely to be closer to the global optima and is actively exploring that area but still doing some minimal exploration (*n* = 45). Source data are provided as a Source Data file

The pathway optimization workflow was implemented using iBioFAB^7^ which has been used for high-throughput TALEN synthesis^48^ and automated yeast genome engineering^49^. By harnessing the power of iBioFAB as well as Bayesian optimization, all aspects of the DBTL cycle were automated. In each round, the Bayesian optimization algorithm chose 46 points to be evaluated (the number being chosen based on tests reported above) and gave them to the iBioFAB scheduling software. As a control and accounting for any variations between different batches, one of the chosen points was always the middle point (12, 12, 12). The software then pipetted the correct parts to be assembled from the parts library and assembled the plasmids using Golden Gate assembly. The lycopene production for the points was then measured in four biological replicates and the mean values of the results were given back to the algorithm to calculate the next points to be evaluated. The Bayesian optimization algorithm starts by uniformly exploring the entire landscape (Fig. 3a) and gets less uniform in the later rounds (Fig. 3b, c) where more information is available about the landscape, prompting exploration of specific regions. In round 2, there is still some exploration while the points in the third round have almost converged to one specific region which is believed to yield the highest lycopene production.

**Fig. 4:**
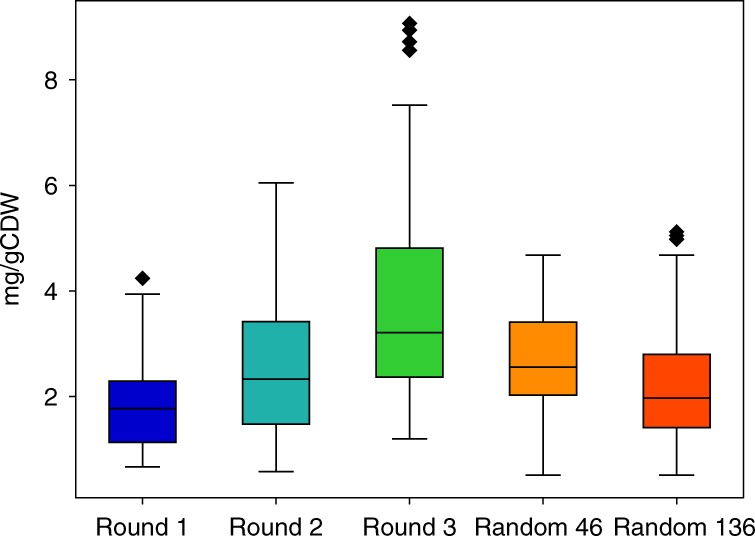
Lycopene production in different rounds of pathway optimization and random screening. The average and maximum points have increased after each round of pathway optimization. Moreover, although the average and maximum of evaluating 46 and 136 random points are a little more than the uniform distribution in round 1, they are significantly lower than the points picked by the algorithm in the subsequent rounds. The boxes of the plots contain data within the interquartile range (IQR), while whiskers spread from the boxes to 1.5 times IQR. The center line in the boxes is the median of the data and points above the whiskers are values which are higher than 1.5 times IQR above the third quartile. *n* = 46, 45, 45, 46 and 136 for each plot, respectively. Source data are provided as a Source Data file

The distributions of lycopene production among points evaluated in each round are compared to each other in Fig. 4, and it is observed that the later rounds of pathway optimization have higher average lycopene production and higher maximum production which shows the effectiveness of the Bayesian optimization algorithm in finding better points (i.e., mutants) in each subsequent round. To better compare the Bayesian optimization algorithm with traditional random library screening, a random library was constructed and 46 (same number as the number of points in each round) and 136 (same number as the number of points in all three rounds) points were randomly picked and lycopene production was measured, and the production distributions of these two collections are also shown in Fig. 4. The average and maximum of lycopene titer found by random screening are 1.43 and 1.93 times less than those found from the third round of pathway optimization. Even by evaluating 136 random points, the maximum of lycopene titer found was 1.77 times lower than the maximum from the third round.

To better compare random sampling with Bayesian optimization and to more reliably represent the maximum found by random sampling, a distribution model was constructed based on the 136 randomly tested points. First, the average and standard deviation for the experimental data were calculated and used to generate a normal distribution. A total of 136 points were randomly selected from this distribution and the maximum was recorded. This was repeated 1000 times and the distribution among maxima is shown in Supplementary Fig. 4. The average and standard deviation of the 1000 maxima dataset was found to be 4.81 and 0.43, respectively, therefore the expected outcome of the best mutant from 136-point random sampling is 4.81 ± 0.43. This simulation is far from perfect because a normal distribution is not necessarily the best representation of the landscape, and because metabolic burden may have reduced the average production amount of randomly selected mutants. Nevertheless, it provides a useful baseline for comparison. The maximum of the 136 points tested in our experiment was 5.12, within the expected outcome calculated. This range is well below 9.07, the best mutant found using the Bayesian optimization method.

The best lycopene producers of each round were also isolated and characterized in test tubes, and lycopene production was quantified using the traditional acetone extraction method^50^. The pathway with the medium-level expression for all genes was also chosen as the control, and the lycopene production levels for all these four samples were analyzed in one batch with four biological replicates and compared to the control (Supplementary Fig. 5). It was observed that the best combination in each round has increased significantly and the best overall lycopene producing strain is eight times better than the control.

## Discussion

In this work, we presented a fully automated algorithm-driven optimization platform for biosystems design where the machine performs all the steps in the optimization process. iBioFAB was integrated with the machine learning algorithm where after the initial design and setup, the algorithm decides what experiments to perform, the robot performs the experiments and returns the data to the algorithm and it will then decide the next point to be evaluated. Machine learning enables exploration of large dimensional optimization problems whereas our intuition is mostly limited to three dimensions. Particularly, machine learning enables faster and more targeted optimization by only focusing on areas of high interest and uncertainty, deals with the experimental data by keeping the uncertainty of experiments into account and actively tries to reduce the number of experiments and the cost. BioAutomata is less biased, can process high-dimensional data, makes fewer mistakes and can find the optimum with very few evaluations.

To demonstrate one of BioAutomata’s applications and as a proof of concept, we set to optimize the flux of the lycopene biosynthetic pathway. We were able to tune the gene expression of this 3-gene pathway to find the optimum expression for the most lycopene production by evaluating <1% of all 13,824 possibilities. We also compared this optimization scheme with another previously reported regression model as well as random sampling and found it to be superior to both in performance. The best mutant found using the BioAutomata produced 1.77-fold higher lycopene titer than the best mutant found using random sampling and simulation showed that the number of evaluations was at least eight times less than the regression-based optimization scheme. The optimization performed here was focused on the intrinsic parameters of the pathway. Through optimization of extrinsic parameters, such as flux control by deleting genes that draw from the pathway or overexpressing genes that feed into the pathway, engineering of the central metabolism, strain optimization or fermentation optimization, higher titers of lycopene expression have been reported in the literature^23^.

The lycopene biosynthetic pathway was specifically chosen in this experiment due to its straightforward methods of extraction and quantification that facilitated high-throughput execution using the automated biofoundry at the time. Potential challenges of a universal application of BioAutomata for pathway optimization include extraction methods that are difficult to perform on an automated platform, or analytical/quantification methods that require equipment more complex than a plate reader, such as Gas Chromatography-Mass Spectrometry (GC-MS) or Liquid Chromatography-Mass Spectrometry (LC-MS) instruments. These challenges can be overcome, but a larger-scale and sophisticated biofoundry must be constructed to integrate these instruments. It is also noteworthy that the promoter characterization in this work was performed with a green fluorescent protein (GFP) gene and not the lycopene biosynthetic genes, and although this assay is a widely used method for promoter/RBS characterization^14,44,45,47,51^, we did not measure the protein expression level of the *crtE*, *crtB* and *crtI* genes in the lycopene biosynthetic pathway which would have resulted in a more accurate mapping of the promoter/RBS sequence and expression level.

Although the algorithm is especially powerful when used in combination with a fully automated system like iBioFAB, it can be easily adopted for use in semi-automated or manual settings where reducing the number of evaluations is even more important due to the higher experimental cost. Moreover, other models and optimization algorithms are available and GP was chosen mainly due to its successful implementation in biological systems^15,16^. An area of possible improvement is the initial guess of the landscape for optimization. Here, we did not make any initial assumptions about the landscape, however, using the trained model for one system as the starting point for a similar system has been shown to be a powerful method and this educated guess can potentially result in reducing the number of evaluations to find the maximum^52^. For instance, the trained posterior on a 3-gene pathway can be used as the starting point for optimizing the same pathway with additional genes to optimize.

This approach and the BioAutomata can be used for other black-box optimization problems where the evaluations are noisy and expensive and are not limited to pathway optimization. One conceivable example can be protein engineering where different changes to the protein sequence can be made using approaches described by Romero and co-workers^15,16^ or using CRISPR-based in vivo point mutation and modification tools^53^ and the optimal change is found using a similar approach. This optimization workflow can also be used in other areas from buffer and media optimization to genome engineering in search for desired phenotypes. Given the highly efficient nature of a 4-piece Golden Gate assembly, it was assumed that all the reactions worked, which may not be a valid assumption in more complex assemblies or optimization systems and an in-line quality control step and an outlier detection method should be added for such complex and error prone systems. We also assumed a uniform noise model in our Bayesian optimization approach for the sake of simplicity. Although this noise model does not match the model used in typical GP regression, we demonstrated that this GP-based Bayesian optimization method was able to operate effectively even with some modest model mismatch. Future applications where variability of measurement noise across experiments is anticipated to be a major concern may find it useful to use heteroskedastic noise models^54^.

The prospect of autonomous algorithm-driven robotic systems for engineering biology has many promises and challenges. On one hand, human supervision is crucial to maintain ethical issues surrounding the autonomous engineering of life and keeping a check on the extent of what the machine does and achieves. On the other hand, an autonomous algorithm-driven robotic system, which is connected to the web of knowledge, can learn from the published information in real-time and publish the results of its experiments in real-time as well. Other than the obvious advantages of reducing the cost and increasing the accuracy of research, the connected web of BioAutomata can significantly reduce the time from performing experiments to publishing the data and using it by others. BioAutomata will greatly benefit from standardization of data and following standards set by databases like Braunschweig Enzyme Database (BRENDA), Kyoto Encyclopedia of Genes and Genomes (KEGG), Protein Data Bank (PDB) and Synthetic Biology Open Language (SBOL)^55–57^.

## Methods

### Strains cultivation

DH5α and BL21(DE3) *Escherichia coli* (New England Biolabs, Ipswich, MA) cells were used for making chemically competent cells using Mix & Go *E. coli* Transformation Kit (Zymo Research, Irvine, CA) for plasmid amplification and lycopene production, respectively. *E. coli* cells were grown in Luria Broth (LB) medium (Fisher Scientific, Pittsburgh, PA) supplemented with 50 μg/mL spectinomycin (Spec) or 25 μg/mL kanamycin (Kan) to maintain the plasmid or 0.5 mM isopropyl-β-d-thiogalactoside (IPTG) for induction as appropriate. Antibiotics and IPTG were purchased from Gold Biotechnology (St. Louis, MO). DH5α *E. coli* cells and BL21(DE3) starter cell cultures were grown at 37 °C, but BL21(DE3) cell cultures for lycopene production were grown at 28 °C, the optimum growth temperature for lycopene production^22,58^. The dry cell weight (DCW) was calculated from the OD_600_ using dcw/OD of 0.36 as the conversion rate^59^.

### DNA manipulation and plasmid construction

To generate the T7 promoter (T7p) variants with different expression levels, the region attributed to its strength^46^ was mutated by using T7p-mut-3N and T7p-mut-6N primers when amplifying eGFP gene with T7 terminator (T7t) primers. The resulting T7p-mut-eGFP-T7t DNA fragment was cloned into the pET26 (b) backbone using restriction digestion ligation. The resulting library was then transformed into BL21(DE3) competent cells and 192 colonies were randomly picked and grown overnight at 37 °C. The next day, 900 μL of LB + Kan was inoculated with 10 μL of the seed culture and was incubated at 37 °C and 250 rpm. After 3 h, 100 μL of LB + Kan with 5 mM IPTG was added to the cell culture and it was incubated at 28 °C. After 4 h, eGFP fluorescence (488 nm excitation/509 nm emission), as well as OD_600_, were measured and 24 different promoters were chosen for further characterization. Two RBS sequences were designed using RBS library calculator^45^ for translation regulation and were combined with the identified promoters to evaluate their strength. These promoters were then used to clone the T7-mut-RBS-eGFP-T7t expression cassette. Twelve of these promoters that exhibited a wide range of strengths were chosen as a promoter library for transcription regulation. To test the expression level of the lycopene genes, the *crtE*, *crtB* and *crtI* genes were PCR amplified and fused to *eGFP* gene and different promoter/RBS combinations using Gibson Assembly. Fusion expression of *crt* genes and *eGFP* was performed with a flexible linker (GGATCCGCTGGCTCCGCTGCTGGTTCTGGCGAATTC) that was optimized for GFP fusion expression in *E. coli*^60^. The T7_mut_RBS(weak/strong)_Crt(E/B/I)_eGFP expression cassettes were then expressed in four biological replicates following the same protocol as above and the fluorescence was measured.

QIAGEN Plasmid Mini Kit (QIAGEN, Valencia, CA) was used to isolate plasmids from *E. coli* cells and Zymoclean Gel DNA Recovery Kit (Zymo Research, Irvine, CA) was used for gel purification. All restriction enzymes, Q5 polymerase, Gibson Assembly master mix components and the *E. coli* shuttle vectors were purchased from New England Biolabs (Ipswich, MA) and all chemicals were purchased from Sigma-Aldrich (St. Louis, MO) unless otherwise specified. All the primers and plasmids used in this study are listed in Supplementary Data 1 and  2, respectively. The GenBank files with the annotated map of DNA parts as well as the final constructs are included in the Supplementary Information. The strains and plasmids are available through the standard material transfer agreement from the University of Illinois.

### Golden Gate assembly

Golden Gate assembly method was used to assemble the lycopene pathway with different expression level of each gene. First, the pSPE plasmid was digested with *AFI*II and *Xba*I restriction enzymes. After digestion, two complementary oligos containing optimized Golden Gate overhangs^48,61^, as well as a T7 promoter, terminator and *EcoR*V recognition site were annealed, phosphorylated and cloned between the cut sites. Phosphorylation was performed using T4 Polynucleotide Kinase (New England Biolabs, Ipswich, MA), following manufacturer’s instructions. The plasmid was then amplified, digested with *EcoR*V and each of the *crtE*, *crtB* and *crtI* genes with different RBS/T7 promoter strengths were cloned using Gibson assembly method^62^ with T7 promoter and terminator as the homology arms by commercial NEBuilder HiFi DNA Assembly Cloning Kit (New England BioLabs, Ipswich, MA) as shown in Supplementary Data 15–18. To create the insert for cloning in the helper plasmids, RBS was added to each of the *crtE*, *crtB* and *crtI* genes using PCR amplification and T7 promoter was added in another step of PCR reaction. The 72 assembled plasmids were then amplified in *E. coli* and the inserts were confirmed by PCR amplification. The pET26b plasmid was obtained from EMD Millipore (Billerica, MA) and used as the receiver for the lycopene biosynthetic pathway. The Golden Gate linkers as well as the *Bsa*I sites were placed on two complementary oligonucleotides resulting a short DNA fragment with sticky end after annealing and phosporylation. pET26b plasmid was digested with *Xho*I and *Sph*I restriction enzymes and ligated to the DNA fragment containing overhangs to construct the backbone for the lycopene production pathway.

The 73 assembled parts (72 inserts and 1 backbone) were amplified in *E. coli* and purified. The concentration of the backbone was set to 30 ng/μL and the concentration of the rest of the parts was adjusted to the same molar concentration. Each 20 μL Golden Gate reaction consists of 100 ng of the backbone, equimolar amounts of *crtE*, *crtB* and *crtI*, 10 units of *Bsa*I restriction enzyme, 100 units of T4 DNA ligase, 2 μL of CutSmart buffer and 0.75 μL of adenosine triphosphate (ATP) (25 mM). After the Golden Gate reaction, 5 μL of nuclease master mix consisting of 2.5 units of *Bsa*I, 2.5 units of plasmid safe nuclease (Illumina, San Diego, CA), 0.5 μL of CutSmart buffer and 1 μL of ATP (25 mM)) was added to the reaction to linearize any undigested backbone and digest all the linear parts from the mixture. The above Golden Gate and plasmid safe master mix protocol have been adopted from our previous work^48^ with some modifications but the thermocycling protocol has remained unchanged. To ensure high-efficiency assemblies, optimized Golden Gate linkers for this experiment were chosen from a highly efficient set of linkers^63^. To test the efficiency and fidelity of Golden Gate assembly, 24 reactions using each of the 72 parts at least once were performed and four colonies from transformants of each reaction were selected and all the assemblies were confirmed to be correct. A sample of these assembly products is shown in Supplementary Data 15.

### Lycopene extraction and quantification

Lycopene can be extracted by organic solvents and quantified calorimetrically by measuring the absorbance at around 470 nm^64^. This assay is highly sensitive and has been reported to quantify the lycopene amount with sub-milligram accuracy^65,66^. The most common lycopene extraction and quantification method involves resuspension of the cells in acetone followed by incubation in acetone^50,67,68^. Since acetone is extremely volatile and dissolves the glue seals and some of the other consumables, it is not ideal for use in the automation system. Therefore, four other organic solvents, some of them were reported in previous publications^22,69^, were tested for efficacy in lycopene extraction. The most effective extraction solvent that is compatible with high-throughput systems was found to be dimethyl sulfoxide (DMSO). *E. coli* cells were spun down and the supernatant was removed. The cells were then resuspended in 300 μL of DMSO and were incubated for 30 min at 37 °C at 250 rpm. After the incubation, the cell–DMSO mixture was spun down at 3000 rpm for 10 min and 200 μL of the supernatant was removed and the absorbance at 472 nm was measured and correlated to lycopene production.

### Full automation of workflow

**Fig. 5:**
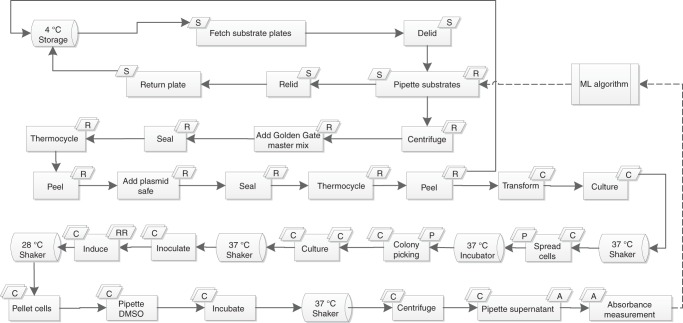
The overall fully automated pathway optimization workflow. The machine learning algorithm picks the plasmids to be assembled and returns the list to iBioFAB to perform the assembly. The assembled products are then transformed, and four single colonies are isolated for lycopene quantification and OD measurement. The resulting data are then given to the machine learning algorithm to pick the next set of points to be evaluated

iBioFAB^7^ was used to automate the assembly of DNA parts for the lycopene pathway, transformation, cell cultivation and lycopene extraction. The overall workflow of the experiments is shown in Fig. 5. First, the parts to be assembled are generated by the machine learning algorithm and given to the previously described^48^ script generator to generate the pipetting routs for the Tecan liquid handler. The DNA mixture plates were then spun down, mixed with Golden Gate master mix and moved to thermocycler for the Golden Gate reaction. After 30 cycles of digestion and ligation in Golden Gate assembly, Plasmid Safe master mix was added to the mix followed by 30 min of digestion with *Bsa*I and plasmid safe nuclease. The plasmid-safe-treated Golden Gate assembly product was then transformed in BL21(DE3) *E. coli* competent cells and plated on LB agar plates and moved off the deck for incubation. The plates were incubated at 37 °C overnight and four single colonies were picked from each of the plates using Pickolo colony-picker (SciRobotics, Israel) and inoculated in 1 mL of LB + Kan media. The seed culture was grown overnight and 50 μL of the culture was added to 800 μL of fresh LB + Kan media and incubated at 37 °C. After 2 h, 200 μL of LB + Kan + 2.5 mM IPTG was added to the culture and the induced cells were incubated at 28 °C for 24 h for maximal production^22^. OD_600_ was measured and the cells were then pelleted and resuspended in DMSO and incubated at 37 °C for 30 min for lycopene quantification. To minimize the possible variations between the different round of optimization, the point with the median expression level of all three genes (12, 12, 12) was repeated in the second and third rounds of optimization. Two other controls for OD (no inoculation and growth) and lycopene production (empty plasmid) in four replicates were included in all three rounds. Therefore, the total number of new points in the first, second and third rounds were 46, 45 and 45, respectively, and each round consisted of two full 96-well plates. To test the efficiency of the assembly, 24 of the assembled combinations were picked at random and were verified with restriction enzyme digestion and all 24 proved to be correct as shown in Supplementary Fig. 6. Three of these 24 plasmids were also sequenced, and the result matched the expected sequence (Supplementary Data 3–14).

### Reporting Summary

Further information on research design is available in the Nature Research Reporting Summary linked to this article.

## Supplementary information

Supplementary Information

Reporting Summary

Description of Additional Supplementary Files

Supplementary Data 1-18

Source Data

## Data Availability

Data supporting the findings of this work are available within the paper and its Supplementary Information files. A reporting summary for this article is available as a Supplementary Information file. The datasets generated and analyzed during the current study are available from the corresponding author upon request. The source data underlying Figs. 3 and 4, Supplementary Figs. 2–5, Table 1, as well as Supplementary Tables 1 and 2 are provided as a Source Data file.

## Code availability

The source code for Bayesian Optimization was obtained from https://github.com/JasperSnoek/spearmint. The code for Exterior Derivative Estimation was obtained from Aswani and coworkers^38^. The pipetting worklist was generated using the program previously described by Chao and coworkers^48^, while the code for running the iBioFAB platform was compiled on the iScheduler scheduling software^48^. All the abovementioned codes and the codes interacting with the Spearmint library are included in the Github: https://github.com/hamedir2/BayesianOptimization. All these pieces of code are provided under the MIT License.
