# Supplementary material for: Towards a fully automated algorithm driven platform for biosystems design
Source: Nat Commun. 2019 Nov 13;10:5150. doi: 10.1038/s41467-019-13189-z (PMC6853954; doi:10.1038/s41467-019-13189-z)
Supplement: Supplementary file 1 — Supplementary Information [file 41467_2019_13189_MOESM1_ESM.pdf]

Towards a fully automated algorithm driven platform for biosystems design

HamediRad *et al.*

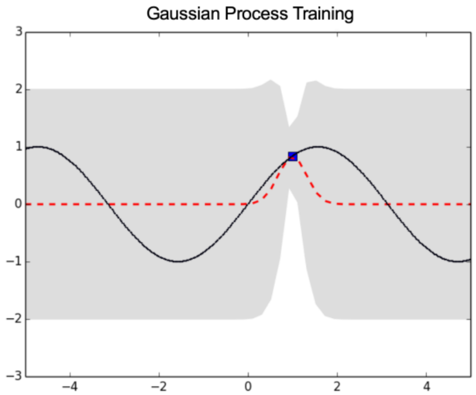

A

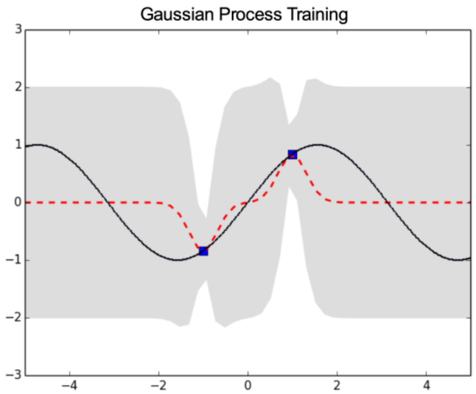

B

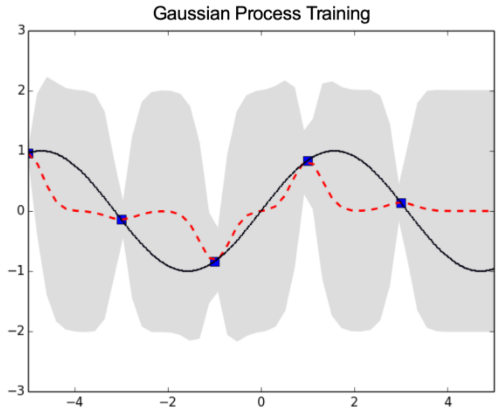

C

Supplementary Figure 1. Demonstration of mean and variance of a Gaussian process estimating a sine function. The dashed lines show the mean and the grey areas two standard deviations from the mean. With only one point evaluated (A), the mean is only slightly adjusted, and the variance has changed around the point evaluated. With two (B) and five (C) points evaluated, the mean gets closer to the sine function and the confidence level increases across the landscape with the most change being near the evaluated points. The mean and variance show the predicted value for those points and the confidence on those predictions respectively. The code for drawing these figures has been borrowed and modified from PMK3 Toolkit (https://github.com/probml/pmtk3).

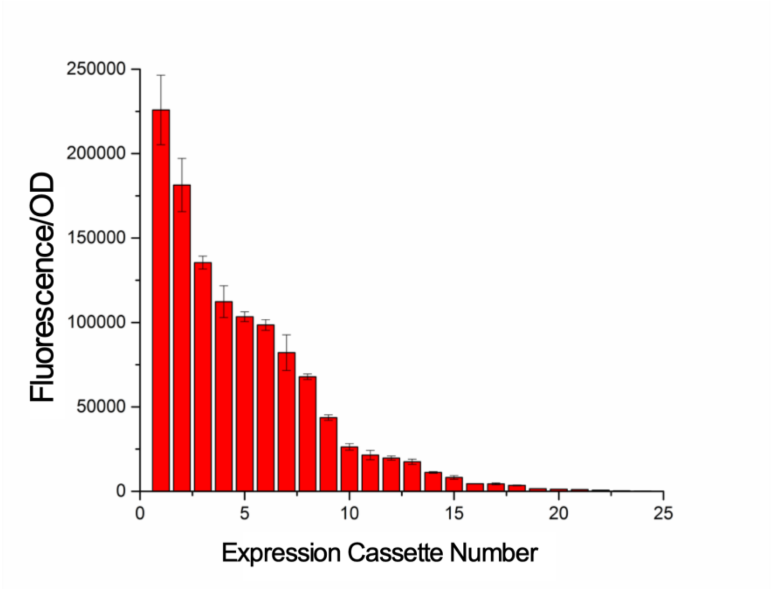

Supplementary Figure 2. Evaluation of 192 T7 promoter mutants and two RBSs resulted in 24 T7p-RBS combinations with ~1000x dynamic range. Error bars represent the mean ± s.d. of biological quadruplicates. Source data are provided as a Source Data file.

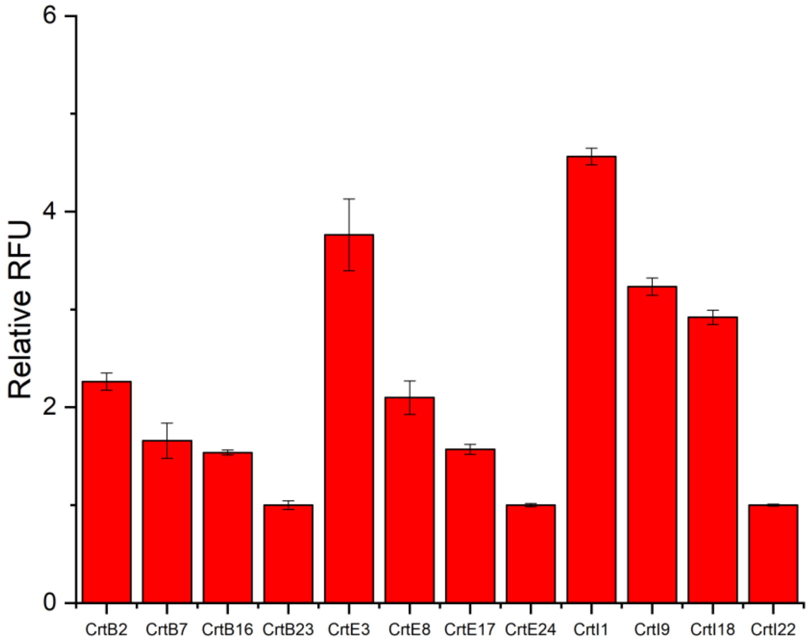

Supplementary Figure 3. Testing the promoter/RBS strength expressing the lycopene pathway genes. *eGFP* was fused to the *crtB*, *crtE*, and *crtI* genes under the control of different promoter/RBS combinations and the florescence was measured. It was observed that the general strength trend is held for the lycopene pathway genes, i.e., for each gene the measured expression levels driven by its four selected promoters are in the order expected based on the pre-determined promoter strength. Error bars represent the mean ± s.d. of biological quadruplicates. Source data are provided as a Source Data file.

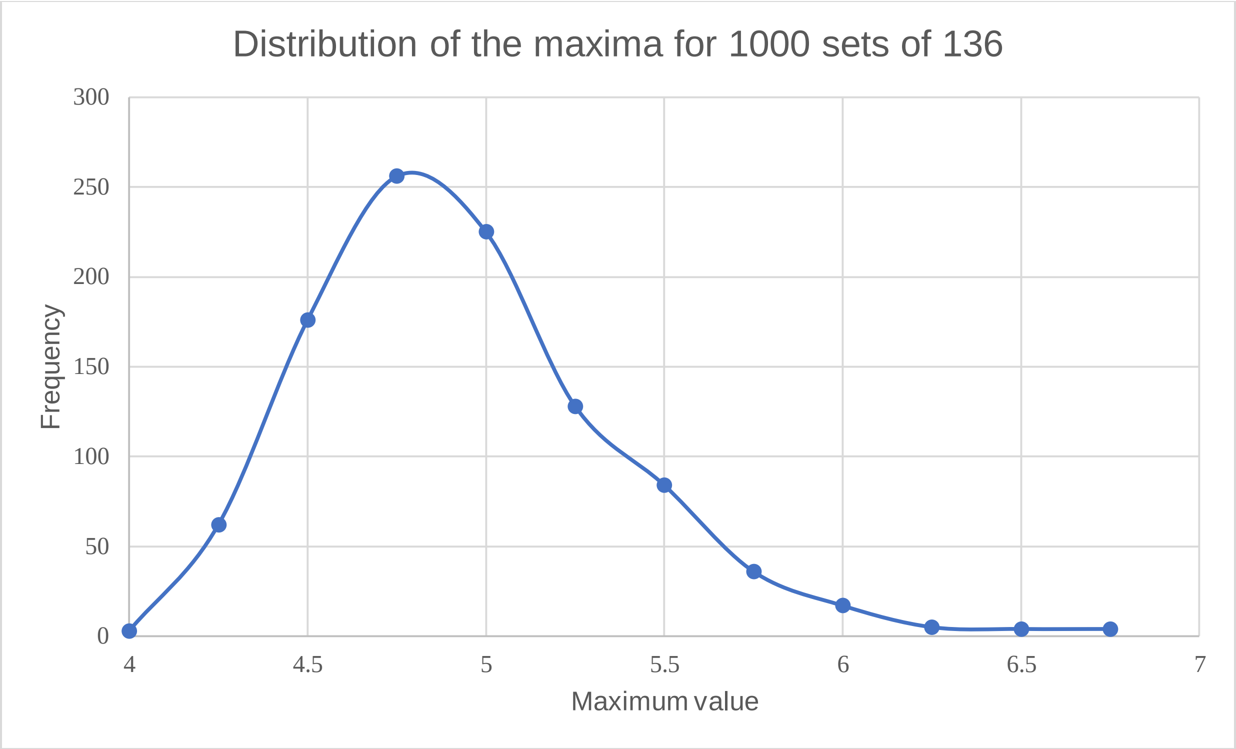

Supplementary Figure 4. Distribution of the maxima for 1000 sets of 136 random samples. Source data are provided as a Source Data file.

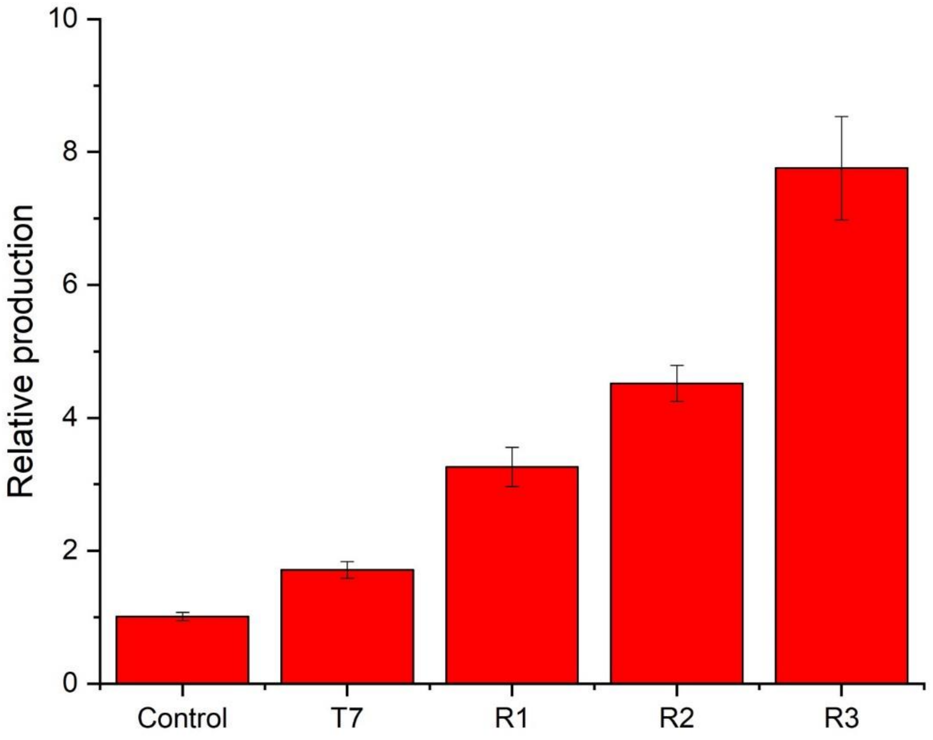

Supplementary Figure 5. Comparison of the best lycopene producers in each round of pathway optimization with the T7 promoter and the middle point as controls. Error bars represent the mean ± s.d. of biological quadruplicates. Source data are provided as a Source Data file.

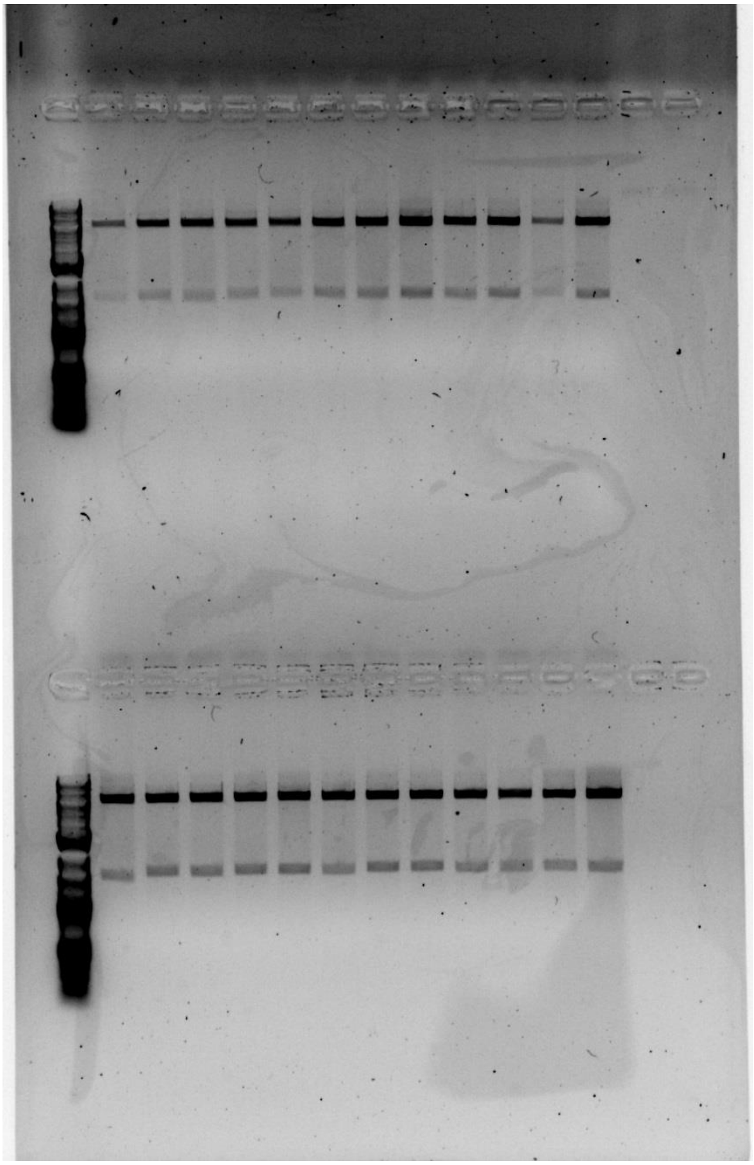

Supplementary Figure 6. Verification of automated Golden Gate assembly via double restriction digestion. 24 Golden Gate assembly reactions were performed on the automated liquid handler and one transformant from each was verified via restriction digestion using *Nco*I-HF and *BamH*I-HF. All digestions showed correct band sizes. Three of these assemblies were also randomly picked and sent for sequencing and all of them matched the expected sequencing pattern.

Supplementary Table 1. Evaluation of a state-of-the-art regression model on a 4-dimensional function.

Number of points	Maximum Predicted	Real value	Maximum
16	6.9	8.2	9
48	7.4	8.4	9
96	7.4	8.4	9
192	7.4	8.4	9

Note: EDE regression model was trained on a different number of randomly picked points and the point with best expected value was predicted by the model and was compared to the real value for that point. Source data are provided as a Source Data file.

Supplementary Table 2. Batch size optimization.

Batch size	Points before max	Batches before max	Points before 95%	Batches before 95%
1	56.81	56.81	22.58	22.58
8	61.20	7.65	20.00	2.50
24	81.84	3.41	39.12	1.63
46	103.96	2.26	63.02	1.37
92	160.77	1.75	105.94	1.15

Note: The batch size was changed from 1 to 92 samples per batch and the number of points before reaching the maximum and 95% of the maximum were compared. All these simulations were repeated 100 times with 10% error and the average was calculated and reported. Source data are provided as a Source Data file.
