# Supplementary material for: Towards a fully automated algorithm driven platform for biosystems design
Source: Nat Commun. 2019 Nov 13;10:5150. doi: 10.1038/s41467-019-13189-z (PMC6853954; doi:10.1038/s41467-019-13189-z)
Supplement: Supplementary file 3 — Description of Additional Supplementary Files [file 41467_2019_13189_MOESM3_ESM.docx]

**Description of Additional Supplementary Files**

File Name: Supplementary Data 1

Description: Primers used in this study

File Name: Supplementary Data 2

Description: Plasmids used in this study

File Name: Supplementary Data 3

Description: Sequencing result 1 from assembly product 1

File Name: Supplementary Data 4

Description: Sequencing result 2 from assembly product 1

File Name: Supplementary Data 5

Description: Sequencing result 3 from assembly product 1

File Name: Supplementary Data 6

Description: Sequencing result 4 from assembly product 1

File Name: Supplementary Data 7

Description: Sequencing result 1 from assembly product 2

File Name: Supplementary Data 8

Description: Sequencing result 2 from assembly product 2

File Name: Supplementary Data 9

Description: Sequencing result 3 from assembly product 2

File Name: Supplementary Data 10

Description: Sequencing result 4 from assembly product 2

File Name: Supplementary Data 11

Description: Sequencing result 1 from assembly product 3

File Name: Supplementary Data 12

Description: Sequencing result 2 from assembly product 3

File Name: Supplementary Data 13

Description: Sequencing result 3 from assembly product 3

File Name: Supplementary Data 14

Description: Sequencing result 4 from assembly product 3

File Name: Supplementary Data 15

Description: pET26_T7_CrtEBI Plasmid Map

File Name: Supplementary Data16

Description: pSPE_CrtB Plasmid Map

File Name: Supplementary Data 17

Description: pSPE_CrtE Plasmid Map

File Name: Supplementary Data 18

Description: pSPE_CrtI Plasmid Map
